# Selective Depletion of Autoreactive Plasma Cells as a Novel Strategy to Treat Acetylcholine Receptor Antibody‐Positive Myasthenia Gravis

**DOI:** 10.1002/eji.70166

**Published:** 2026-03-29

**Authors:** Laleh Khodadadi, Deborah Puppe, Dilara S. Cirillo, Carolina Martinez‐Cingolani, Jens Klotsche, Andreas Pelz, Siegfried Kohler, Qingyu Cheng, Konstantinos Lazaridis, Michael Fichtner, Jan Pille, Andrey Kruglov, Marina Bondareva, Tobias Alexander, Andreas Radbruch, Andreas Meisel, Falk Hiepe

**Affiliations:** ^1^ Department of Rheumatology and Clinical Immunology Charité – Universitätsmedizin Berlin, Corporate Member of Freie Universität Berlin and Humboldt‐Universität zu Berlin Berlin Germany; ^2^ German Rheumatology Research Center (DRFZ) Leibniz Institute Berlin Germany; ^3^ Sanofi R&D, Immunology and Inflammation Therapeutic Research Area Vitry‐sur‐Seine France; ^4^ Department of Neurology with Experimental Neurology Charité – Universitätsmedizin Berlin, Corporate Member of Freie Universität Berlin and Humboldt‐Universität zu Berlin Berlin Germany; ^5^ Department of Neurology Sana Klinik Biberach Biberach Germany; ^6^ Department of Immunology Hellenic Pasteur Institute Athens Greece; ^7^ Max Delbrück Center For Molecular Medicine MDCell Helmholtz Innovation Lab Berlin Germany; ^8^ Neuroscience Clinical Research Center Charité – Universitätsmedizin Berlin, Corporate Member of Freie Universität Berlin, Humboldt‐Universität zu Berlin Berlin Germany; ^9^ Center for Stroke Research Berlin Charité – Universitätsmedizin Berlin, Corporate Member of Freie Universität Berlin, Humboldt‐Universität zu Berlin Berlin Germany

**Keywords:** autoreactive plasma cells, autoimmunity, myasthenia gravis, selective depletion

## Abstract

Myasthenia gravis (MG) is a chronic autoimmune disease mediated by autoantibodies targeting the neuromuscular junction and leading to muscle weakness. Although there are autoantibodies of different specificities, most MG patients have autoantibodies directed against the nicotinic acetylcholine receptor (AChR), in particular against the extracellular domain of the α1 subunit (αECD), containing the main immunogenic region (MIR). Here, we demonstrate an original approach to selectively deplete plasma cells secreting autoantibodies targeting αECD. An antibody‐mediated cytotoxicity‐engager (ACE) consisting of an anti‐hCD38‐antibody conjugated to hAChR αECD (αECD) was used to deplete hAChR αECD‐specific cells selectively in vivo. The reduction of pathogenic cells was accompanied by lower antibody titers, a reduction of MG disease score, protection of grip strength, and maintenance of body weight. Notably, antibody‐secreting cells that are nonspecific for hAChR αECD were not affected. The resulting amelioration of MG pathology in ACE‐treated animals highlights the decisive role of αECD‐antibodies in the pathogenesis of MG and the clinical relevance of the novel therapeutic strategy.

## Graphical abstract

Current plasma cell–targeting therapies lack antigen specificity. Using an antibody‐mediated cytotoxicity engager (ACE) targeting the α‐subunit of the extracellular domain of the human acetylcholine receptor (hAChR αECD), we selectively depleted pathogenic antibody‐secreting cells in a myasthenia gravis model, reducing autoantibody titers and disease severity while preserving nonspecific antibody‐secreting cells and humoral immunity.

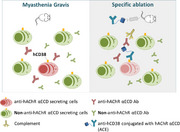

**Abbreviations**
αECD: extracellular domain of the α1 subunit of the acethylcholine receptor
βECD: extracellular domain of the β subunit of the acethylcholine receptor
AChR: acetylcholine receptor
anti‐hCD38/αECD‐ACE: anti‐hCD38/αECD antibody‐mediated cytotoxicity engager
ASC: antibody‐secreting cell
B: biotin
hCD38‐B3/2: human CD38 transfected into mouse anti‐human AChR βECD secreting B3/2 (GFP^+^) hybridoma cells
hCD38‐D6: human CD38 transfected into mouse anti‐human AChR ⍺ECD secreting D6 (GFP^−^) hybridoma cells
IgG: immunoglobulin G
LLPC: long‐lived plasma cell
MIR: main immunogenic region
MG: myasthenia gravis
NSG‐Hc^1^
: NOD.Cg‐*Hc^1^ Prkdc* ^scid^ *Il2rg^t^* ^m1Wjl^/*SzJ* mice
SA: streptavidin

## 1 Introduction

Myasthenia gravis (MG) is a prototypic antibody‐mediated autoimmune disease that affects the signal transmission at the neuromuscular junction (NMJ) and is characterized by skeletal muscle fatigability and weakness [1]. In approximately 85% of patients, the main target of autoantibodies is the acetylcholine receptor (AChR). Less common autoantigens include the muscle‐specific kinase (MuSK) and the low‐density lipoprotein receptor‐related protein 4 (LRP4) [2]. The AChR is a pentameric transmembrane protein with a stoichiometry of α_2_βδε in adults [3]. Binding of antibodies to the extracellular domains leads to steric inhibition of the ligand‐binding site between the subunits αε and αδ, antigenic modulation, and complement‐dependent degradation of the muscle end‐plate [2]. The extracellular domain of the α‐subunit (αECD) has a predominant role in MG pathogenesis, as it contains the main immunogenic region (MIR) at the amino acid residues 67–76 [4] and is recognized by two‐thirds of the AChR‐specific antibodies [5]. Its accessible position toward the synaptic cleft and the occurrence of two MIRs per receptor predispose this subunit for effective antibody binding and subsequent antigenic modulation, explaining the immunogenic potential of the alpha subunit [6]. A recent meta‐analysis pointed to a positive correlation between autoantibody levels and disease activity in patients with MG [7]. Nevertheless, it remains controversial whether antibody titres can guide treatment decisions. Anti‐MIR antibody titers have been suggested to be a predictor of disease severity [8]. These data suggest a crucial role for AChR αECD‐antibodies in MG pathogenesis, making them and the cells secreting them a potential therapeutic target.

MG has traditionally been considered a chronic disease requiring lifelong immunosuppressive treatment. State‐of‐the‐art, MG patients are treated by thymectomy, with acetylcholinesterase inhibitors, corticosteroids, immunosuppressants, and plasmapheresis, all therapies being symptomatic [1]. Therapies targeting complement, neonatal Fc receptor, and generically all B‐ and plasma cells are more advanced therapeutic strategies [9]. Targeting plasma cells with anti‐CD38 antibodies, for example, daratumumab, has shown promising results in antibody‐mediated rheumatic and neurological disorders [10]. However, this generic ablation of plasma cells also ablates protective plasma cells, induces loss of humoral memory, and immunodeficiencies [10]. More recently, CD19‐directed CAR‐T cell therapy has induced sustained remission in patients with refractory MG [11].

A novel strategy for selective ablation of plasma cells according to the specificity of the (pathogenic) antibodies they secrete is described here. We use antibody‐mediated cytotoxicity engagers (ACEs) to decorate plasma cells with the antigen‐of‐interest *in vivo*. Plasma cells secreting specific antibodies will be ablated by complement‐mediated and cellular cytotoxicity targeted to them by their own antibodies, relocated to their surface after secretion [12, 13].

Here, we demonstrate the feasibility of this concept for the treatment of MG, with an ACE consisting of a CD38‐specific antibody conjugated to the αECD of the AChR, in a humanized MG mouse model. Selective ablation of αECD‐specific antibody‐secreting cells is sufficient to ameliorate MG symptoms significantly in this model.

## 2 Results

### 2.1 Transfer of Anti‐αECD Antibody‐Producing Cells Induces MG‐Like Symptoms in NSG‐Hc^1^ Mice

**FIGURE 1:**
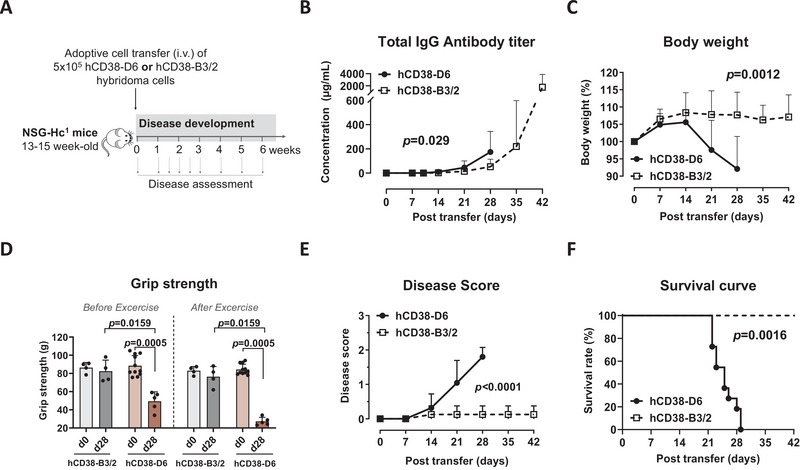
Humanized mouse model of anti‐AChR antibody‐positive generalized myasthenia gravis (NSG‐Hc1 MG mice). (**A**) Animal experiment design. Immunodeficient NSG‐Hc1 mice were divided into two groups, and 5 × 10^5^ hCD38‐D6 (GFP^−^) (*n* = 11) or hCD38‐B3/2 (GFP^+^) (*n* = 4) hybridoma cells were transferred intravenously. Induction of NSG‐Hc1 MG mice was assessed by (**B**) anti‐IgG antibody concentrations secreted by the hybridoma cells (µg/mL), (**C**) body weight (%), (**D**) grip strength (g), (**E**) disease score, and (**F**) survival of each group. Values are shown as mean ± SD and include only the animals alive at the respective timepoints. Statistical analyses were performed using the Mann–Whitney *U* test (D), restricted maximum likelihood analysis (B, C, E), or the log‐rank test (F). As reflected in the survival curve (F), several hCD38‐D6 mice reached predefined endpoints and were euthanized on day 22 (*n* = 3), day 23 (*n* = 2), day 25 (*n* = 2), day 26 (*n* = 1), day 28 (*n* = 1), and day 29 (*n* = 2) in accordance with the endpoint protocol approved by LAGeSo. Accordingly, the values shown represent only the animals alive at each respective timepoint. hCD38‐D6, human CD38 transfected into mouse anti‐human AChR ⍺ECD secreting D6 (GFP^−^) hybridoma cells; hCD38‐B3/2, human CD38 transfected into mouse anti‐human AChR βECD secreting B3/2 (GFP^+^) hybridoma cells.

To establish a humanized MG mouse model, we intravenously transferred 5 × 10^5^ hCD38‐D6 (hD6) hybridoma cells secreting anti‐hAChR αECD antibodies, or hCD38‐B3/2 (hB3/2) hybridoma cells secreting anti‐hAChR βECD antibodies, into immunodeficient NSG‐Hc^1^ mice (Figure 1A). NSG‐Hc^1^ is an immunodeficient derivative of NSG (NOD‐scid IL2rγ^null^) mice, which nevertheless still have an intact complement system [14].

Engraftment of transferred cells was determined according to expression of anti‐αECD or anti‐βECD IgG antibodies in the recipient mice, which developed increasing titers of total IgG antibodies over time, deriving from the respective hybridoma cell line (Figure 1B). In mice transferred with anti‐hAChR αECD antibody‐secreting hCD38‐D6 cells, but not in those transferred with hB3/2 cells, this increase was accompanied by a significant loss of body weight and grip strength and an increase in disease score and lethality between days 21 and 28 posttransfer (Figure 1C–F). Due to the severity of the disease development in mice receiving hD6 cells, some mice died or had to be sacrificed (Figure 1F). These results confirm the successful engraftment of the mice with hD6 and hB3/2 hybridoma cells, and the pathogenic role of the anti‐αECD antibodies secreted by hD6, which apparently suffice to induce MG‐like symptoms in the mice.

### 2.2 In Vitro and in Vivo Labeling of Cells by Anti‐hCD38/αECD‐ACE

**FIGURE 2:**
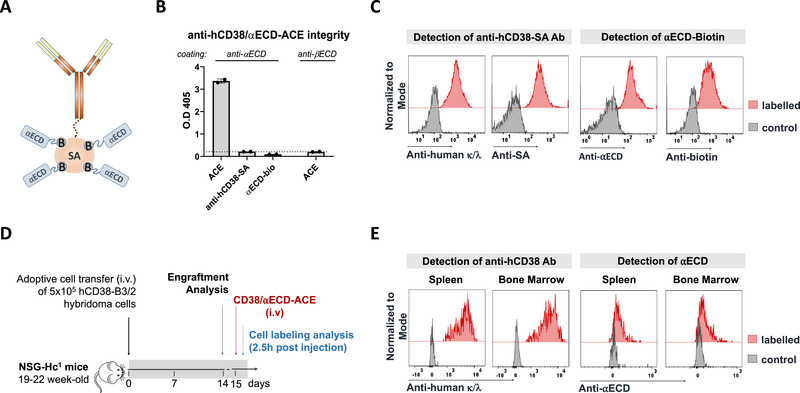
Integrity assessment of anti‐hCD38/⍺ECD‐ACE coupled by SA/biotin. (A) Schematic structure of anti‐hCD38/⍺ECD‐ACE coupled by SA/Biotin. Integrity analysis of anti‐hCD38/⍺ECD‐ACE confirmed by: **(B**) ELISA via coating with anti‐αECD (D6) and anti‐βECD (B3/2) antibodies and detection with anti‐human IgG and **(C)** in vitro labeling of 2 × 10^5^ hCD38‐D6 cells with 5 µg anti‐hCD38/⍺ECD‐ACE at a final concentration of 50 µg/mL and detection of both components of antibody (anti‐hCD38 and SA) and antigen (⍺ECD and biotin) by flow cytometry. **(D)** Experimental design to demonstrate the in vivo labeling of hCD38‐B3/2 (GFP^+^) cells by intravenous injection of 200 µg anti‐hCD38/⍺ECD‐ACE. **(E)** Histogram graphs representing the in vivo labeling of cells and detection of antibody and antigen parts in both spleen and bone marrow of hCD38‐B3/2 (GFP^+^) transferred into NSG‐Hc^1^ mice (*n* = 3 per group). anti‐hCD38/⍺ECD‐ACE, anti‐hCD38/⍺ECD‐antibody‐mediated cytotoxicity engager; hCD38‐B3/2, human CD38 transfected into mouse anti‐human AChR βECD secreting B3/2 (GFP^+^) hybridoma cells.

In order to target cells secreting anti‐αECD antibodies selectively, we designed an anti‐hCD38/αECD antibody‐mediated cytotoxicity engager (anti‐hCD38/αECD‐ACE) (Figure 2A), displaying hCD38‐expressing cells with the αECD antigen for ablation by αECD‐specific antibody‐mediated cytotoxicity. Integrity of the anti‐hCD38/αECD‐ACE molecules was confirmed by ELISA. Plates were coated with human αECD‐specific D6 antibodies to capture the αECD domain of the anti‐hCD38/αECD‐ACE. Bound anti‐hCD38 was detected with anti‐human IgG antibodies. The individual components, anti‐hCD38/SA and αECD/biotin, alone did not show a signal in ELISA. Human βECD‐specific B3/2 antibodies as a coat did not bind the anti‐hCD38/αECD‐ACE (Figure 2B). The disease induction was confirmed in a second, independent experiment using the parental NSG strain (Figure S3).

The anti‐hCD38/αECD‐ACE was able to label hCD38‐positive D6 cells in vitro. All components, that is, the hCD38‐specific antibody, streptavidin (SA), the αECD domain, and biotin, were detectable at the surface of the hCD38‐expressing cells after in vitro labeling of 2 × 10^5^ cells with 5 µg anti‐hCD38/αECD‐ACE at a final concentration of 50 µg/mL (Figure 2C). In vivo labeling of hCD38^+^ cells with the anti‐hCD38/αECD ACE was tested on engrafted hCD38^+^ GFP^+^ B3/2 hybridoma cells, 15 days after transfer into NSG‐Hc^1^ mice. Cells were isolated 2.5 h after intravenous injection of 200 µg anti‐hCD38/αECD‐ACE and stained for the components of the ACE (Figure 2D). Anti‐hCD38 and αECD antigen could be detected on GFP^+^ hB3/2 cells isolated from the bone marrow or spleen of the mice at comparable levels to the in vitro labeling (Figure 2E), confirming the integrity and binding of the anti‐hCD38/αECD‐ACE in vivo.

To assess whether the injection of αECD as part of the ACE might result in a titer reduction in humanized NSG‐Hc^1^ MG mice, the mice received 50, 100, or 200 µg of the autoantigen intravenously (Figure S4A). A short‐term reduction of the antibody concentration was observed 2 h after application of 100 and 200 µg αECD; however, the titers fully recovered within 24 h (Figure 4B).

### 2.3 Selective Depletion of Cells Secreting αECD‐specific Antibodies in Vivo Prevents Development of MG‐Like Symptoms

**FIGURE 3:**
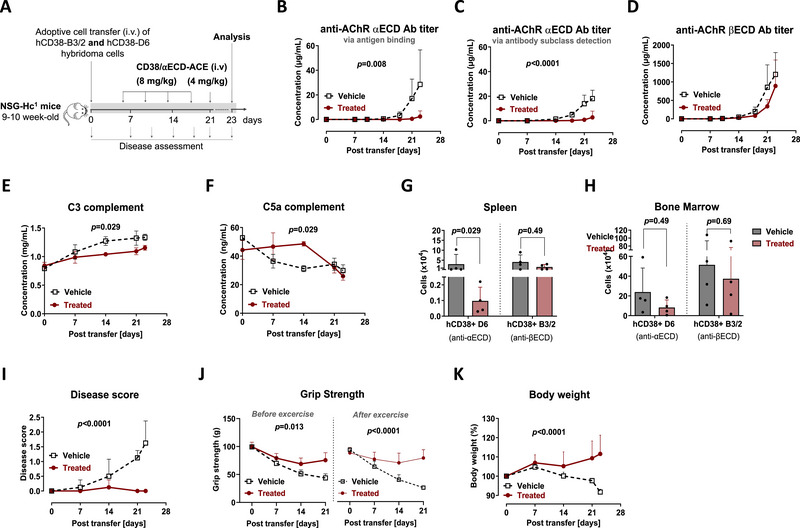
Selective depletion of ⍺ECD‐specific hybridoma cells using anti‐hCD38/⍺ECD‐ACE in humanized NSG‐Hc ^1^ MG mice. (A) Animal experiment design: Immunodeficient NSG‐Hc^1^ mice received 4 × 10^5^ of hCD38‐B3/2 (GFP^+^) and 1 × 10^5^ hCD38‐D6 (GFP^−^) cells intravenously. Mice were divided into two groups: vehicle group (*n* = 4) and treated group (n=4), which received intravenous injections of 8 mg/kg anti‐hCD38/⍺ECD‐ACE twice per week for 2 weeks, followed by a decreased dose of 4 mg/kg for the final treatment before analysis, which took place 2 days after the last treatment. Treatment effect was assessed by serum level of **(B)** IgG anti‐⍺ECD‐antibodies, **(C)** IgG2b anti‐⍺ECD‐antibodies, **(D) IgG** anti‐βECD antibodies, **(E)** complement C3, and **(F)** complement C5a. Flow cytometry analysis on day 23 was used to analyze cell numbers of hCD38^+^GFP^+^ (βECD‐specific B3/2 cells) and hCD38^+^GFP^−^ (⍺ECD‐specific D6 cells) in **(G)** spleen and **(H)** bone marrow of ACE‐ and vehicle‐treated mice. Disease outcome was assessed via **(I)** disease score, **(J)** grip strength test (in grams), and **(K)** body weight (%). Statistics: two‐way ANOVA for (B–G), (I–K) (analysis on day 14) and Mann–Whitney *U* test for (G) and (H), values are mean ± SD. hCD38‐D6; human CD38 transfected into mouse anti‐human AChR ⍺ECD secreting D6 (GFP^−^) hybridoma cells, hCD38‐B3/2; human CD38 transfected into mouse anti‐human AChR βECD secreting B3/2 (GFP^+^) hybridoma cells, anti‐hCD38/⍺ECD‐ACE: anti‐hCD38/αECD‐antibody‐mediated cytotoxicity engager.

**FIGURE 4:**
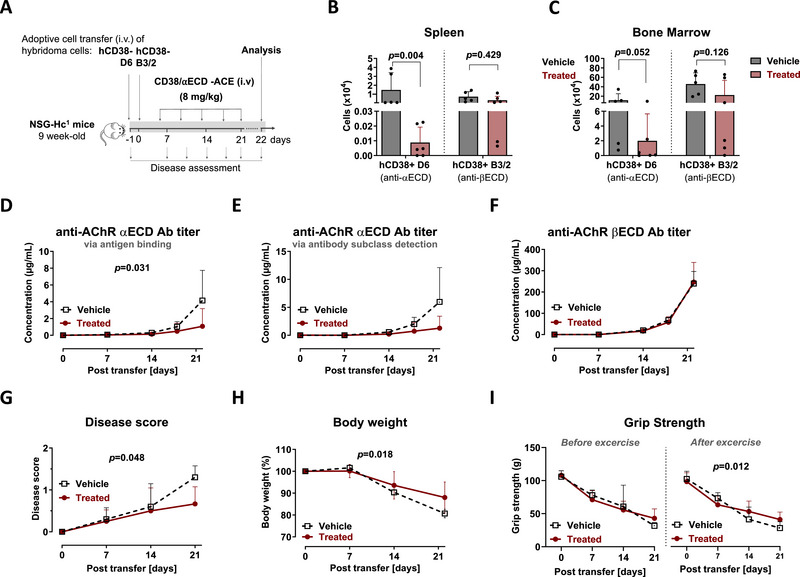
Repetition of the selective depletion of ⍺ECD‐specific hybridoma cells using anti‐hCD38/⍺ECD‐ACE in humanized NSG‐Hc^1^ MG mice. (A) Animal experiment design: Immunodeficient NSG‐Hc^1^ mice received 1.5 × 10^5^ hCD38‐D6 (GFP^−^), and the next day 6 × 10^5^ of hCD38‐B3/2 (GFP^+^) cells intravenously. Mice were divided into two groups: vehicle group (n=5) and treated group (n=6), which received intravenous injections of 8 mg/kg anti‐hCD38/⍺ECD‐ACE twice per week for two and a half weeks. Flow cytometry analysis on day 22 was used to analyze cell numbers of hCD38^+^GFP^+^ (βECD‐specific B3/2 cells) and hCD38^+^GFP^−^ (⍺ECD‐specific D6 cells) in **(B)** spleen and **(C)** bone marrow of ACE‐ and vehicle‐treated mice. Treatment effect was assessed by serum level of **(D)** IgG anti‐⍺ECD antibodies, **(E)** IgG2b anti‐⍺ECD antibodies, **(F)** IgG anti‐βECD antibodies, **(G)** disease score, **(H)** body weight (%), and **(I)** grip strength test (in grams). Statistics: Mann–Whitney *U* test for (B) and (C), two‐way ANOVA for (D–I), values are mean ± SD. anti‐hCD38/⍺ECD‐ACE; anti‐hCD38/⍺ECD‐antibody‐mediated cytotoxicity engager; hCD38‐D6; human CD38 transfected into mouse anti‐human AChR ⍺ECD secreting D6 (GFP^−^) hybridoma cells, hCD38‐B3/2; human CD38 transfected into mouse anti‐human AChR βECD secreting B3/2 (GFP^+^) hybridoma cells.

The treatment with anti‐hCD38/αECD‐ACE was evaluated in two independent in vivo experiments (Figures 3 and 4), conducted in accordance with German animal welfare legislation and the 3R principles. The overall study design was consistent across both experiments, with minor refinements in the second experiment to meet endpoint requirements and to optimize the injection procedure by separating hB3/2 and hD6 cells. In the first experiment, hB3/2 (4 × 10^5^) and hD6 (1 × 10^5^) hybridoma cells were co‐transferred intravenously into NSG‐Hc^1^ mice. Both cell types displayed comparable antibody secretion rates and CD38 expression (Figure S1A,B). Six days after transfer, mice received anti‐hCD38/αECD‐ACE in PBS (8 mg/kg, i.v.) twice weekly for 2 weeks, followed by a final dose (4 mg/kg, i.v.) 2 days before study termination (Figure 3A). Serum concentrations of αECD‐ and βECD‐specific antibodies were monitored over time in ACE‐treated and control groups. Anti‐βECD antibody titers did not differ significantly between groups (Figure 3D). In contrast, ACE‐treatment significantly suppressed the development of anti‐αECD antibody titers (Figure 3B,C). These findings indicate that antibody‐secreting cell ablation was selective for hD6 cells, while hB3/2 cells remained unaffected. Ablation of hD6 cells was further associated with complement activation, as observed by a significant reduction in serum C3 and increased C5a levels, indicative of C5 activation (Figure 3E,F).

To directly assess the selective ablation of hD6 cells versus hB3/2 cells, splenocytes were isolated on day 23 post‐transfer, 2 days after final treatment, and analyzed ex vivo. Both cell types (hD6 and hB3/2) were gated based on human CD38 expression, with hB3/2 cells further distinguished from hD6 by GFP expression (Figure S2). ACE‐treatment resulted in >90% depletion of hD6 cells in the spleen, whereas hB3/2 cell numbers were not significantly affected (Figure 3G).

In the bone marrow, hD6 cell numbers were reduced by 66%, but this did not represent significant depletion, likely reflecting the tenfold higher engraftment compared with the spleen. In addition, insufficient labeling by anti‐hCD38/αECD‐ACE, resulting from rapid proliferation of cells within 2 days after the final injection, may also have contributed (Figure 3H).

In line with the significantly lower anti‐αECD antibody titers in ACE‐treated mice, these mice preserved grip strength, showed no significant disease manifestations, and gained body weight compared with untreated controls (Figure 3I–K). Together, these findings indicate that selective ablation of αECD‐specific antibody‐secreting cells by the αECD‐ACE effectively eliminated the pathogenic driver of MG pathology.

The treatment was repeated in a second, independent experiment designed to resolve the issue of hybridoma clustering and bystander depletion; hB3/2 and hD6 cells were injected separately (Figure 4A). Mice were treated with anti‐hCD38/αECD‐ACE (8 mg/kg, i.v., twice weekly) and sacrificed 1 day after the final dose. This modified approach improved depletion of hCD38^+^ D6 cells in both spleen and bone marrow compared with the initial trial (Figure 4B,C). However, a higher fraction of hCD38^−^ D6 cells engrafted in this experiment (Figure S1B,C), expanded exponentially, and escaped targeting, resulting in low but detectable anti‐αECD antibody titers despite treatment (Figure 4D,E), while anti‐βECD remained unaffected (Figure 4F). ACE treatment improved disease score and body weight (Figure 4G,H), but residual anti‐αECD titers correlated with mild pathology, including weight loss and reduced grip strength (Figure 4I), consistent with disease induction by hCD38^−^ D6 cells.

## 3 Discussion

In this study, we could show for the first time the efficacy of selectively depleting pathogenic plasma cells in an antigen‐specific way as a novel therapeutic strategy for autoantibody‐mediated diseases, using a humanized mouse model of MG. Long‐lived plasma cells (LLPCs), which continuously secrete pathogenic autoantibodies and resist conventional immunosuppressive drugs and B‐cell‐targeted therapies [15], have become a primary focus for therapeutic intervention in antibody‐mediated diseases. Several strategies have been explored, including immunoablation with antithymocyte globulin followed by autologous stem cell transplantation [1], proteasome inhibitors such as bortezomib [9], or T cell engagers [16]. Generic targeting of plasma cells with anti‐CD38 monoclonal antibodies has also shown efficiency in neurological autoimmune diseases, including MG [1, 10]. More recently, CAR‐T cells directed against BCMA have been tested in MG‐patients, leading to persistent depletion of pathogenic autoantibodies and clinical improvement [17]. However, these approaches lack the selectivity of autoreactive plasma cells and carry the risk of humoral immunodeficiency with the need for immunoglobulin substitution. Other targeted therapies, such as complement C5 inhibitors and neonatal Fc receptor inhibitors, have been used in MG patients [9], but their specificity remains insufficient. An alternative approach using CAR‐T cells engineered to recognize autoantigen‐specific B cells has shown promising results in mouse models of autoimmune diseases like NMDAR encephalitis [18]. Although this method specifically targets specific B cells, it does not address LLPCs.

To overcome this limitation and enable targeting of autoreactive LLPCs without broadly ablating plasma cells, we developed a therapeutic strategy that allows depletion of plasma cells secreting pathogenic autoantibodies in an (auto)antigen‐specific manner [12, 13]. To test this concept, we generated a humanized mouse model of MG and demonstrated the feasibility of this innovative therapeutic approach. Hybridoma cells specific to the αECD and βECD of the AChR, engineered to express hCD38, were co‐transferred into immunodeficient NSG‐Hc^1^ mice. The monoclonal antibody D6 binds to residues 65–78 of the human αECD, a region within the main immunogenic region (MIR) [19], and crossreacts with mouse AChR in vivo [20], leading to receptor loss and disease symptoms. Adoptive transfer of anti‐αECD secreting cells induced MG‐like symptoms, including weight loss, reduced grip strength, elevated serum antibody titers, and disease scores. In contrast, transfer of βECD‐specific cells (clone B3/2) did not induce symptoms despite rising antibody titers against βECD. This effect may not solely be attributed to the affinity of anti‐αECD antibodies, but also to their IgG2b isotype, which strongly activates complement in mice, while βECD‐specific antibodies are of the weakly complement‐fixing IgG1 isotype.

Treatment with the anti‐hCD38/αECD‐antibody‐mediated cytotoxicity engager effectively prevented disease onset in NSG‐Hc^1^‐MG mice, maintaining a symptom‐free state in the treated animals, whereas vehicle‐treated mice developed significant weight loss and worsening disease scores. Anti‐hCD38/αECD‐ACE‐treatment significantly reduced anti‐αECD antibody titers while leaving anti‐βECD titers unaffected, demonstrating selective depletion of anti‐αECD‐secreting hybridoma cells. A slight increase in anti‐αECD titers by day 23 likely reflected persistence of hCD38^−^ hybridoma cells or regrowth of residual hCD38^+^ cells after reduced dosing. Flow cytometric analysis confirming selective depletion of hD6 cells with minimal impact on hB3/2 cells, likely due to the clustering of these hybridoma cells. Residual hD6 cells persisted either as nonsecreting variants or after downregulation of hCD38, rendering them refractory to hCD38/αECD‐ACE and capable of inducing mild disease. This effect was especially seen in the repeat experiment, where injected D6 cells had a higher proportion of CD38^−^ cells, highlighting a limitation of the hybridoma‐based induction model. By contrast, naturally occurring plasma cells stably express CD38 and form only loose aggregates in the bone marrow [21], reducing the risk of persistent autoreactive LLPC off‐target effects on protective immunity.

In MG, autoantibodies secreted by both short‐ and long‐lived plasma cells against the AChR play a central role in leading to impaired signal transduction and subsequent muscle weakness and fatiguability [1, 2]. Although anti‐AChR antibody levels do not always correlate with disease severity, clinical improvement is often linked to reduced antibody levels [22]. Antibodies against αECD of the human AChR are particularly potent in inducing MG in animal models [23]. This effect likely results from the high immunogenicity of this domain, especially the main immunogenic region (MIR) [4, 24]. It has also been shown that intravenous administration of a soluble form of αECD alone effectively suppressed ongoing experimental autoimmune myasthenia gravis (EAMG) in rats via tolerization [25]. In this context, it is conceivable that the anti‐hCD38/αECD‐ACE may contribute to the reduction in measured autoantibody titers not only through the demonstrated depletion of autoreactive ASCs but also, to a limited extent, by neutralizing circulating antibodies via its antigen moiety. However, the short‐term antigen‐injection experiments presented here indicate that masking alone results only in a brief and minimal reduction in serum autoantibody levels and does not reproduce the sustained clinical benefit observed with plasma cell targeting. Together, these results underscore the potential of αECD‐targeted therapies in managing autoimmune diseases by specifically addressing pathogenic plasma cells and reducing pathogenic antibody levels of accessibility for antibody‐mediated cytotoxicity.

When compared with existing plasma cell–directed therapeutic strategies, the present approach differs substantially in both target specificity and breadth of plasma cell depletion. CD19‐directed therapies have been shown to ablate approximately 50% of plasma cells, predominantly those derived from extrafollicular and chronic secondary immune responses, while sparing long‐lived CD19^−^ plasma cells generated during germinal center reactions [26]. In contrast, the autoantigen‐directed strategy described here enables depletion of both CD19^+^ and CD19^−^ pathogenic plasma cells. BCMA‐targeting strategies, such as the bispecific antibody teclistamab, induce rapid and profound plasma cell depletion. However, they also result in broad ablation of the B‐cell lineage and plasma cell compartment, frequently leading to humoral immunodeficiency and necessitating immunoglobulin replacement therapy [16, 27]. By contrast, the present approach is designed to selectively eliminate autoantigen‐specific plasma cells while preserving nonpathogenic plasma cells and protective humoral immunity, supporting the potential of this strategy as a precision alternative to lineage‐wide plasma cell ablation.

Since each model can only cover certain aspects of a human disease, we acknowledge several limitations of the study. First, it is notable that the efficacy of our treatment may be influenced by the subclass of the autoantibodies. Therefore, the use of monoclonal antibodies from hybridoma cells may not fully represent the polyclonal antibody responses in MG patients. However, in MG, anti‐AChR antibodies are predominantly IgG1 and IgG3, which are potent activators of the human complement system, which is why C5‐complement inhibition is highly effective [9]. Therefore, this therapeutic approach can be considered quite potent in AChR‐MG. Secondly, treatment was initiated early (day 6) due to the rapid and exponential proliferation of hybridoma cells, which might not accurately reflect patient disease progression. As our mouse model does not fully replicate human autoimmune processes, the rapid growth of hybridoma cells in mice versus the slower autoimmune cell generation in humans may affect treatment timing and frequency.

Since our therapeutic approach is restricted to plasma cells without targeting their progenitor B cells, there is a potential risk for the recurrence of new autoreactive plasma cells when autoreactive B cells are reactivated. To prevent this, a combinatorial approach targeting autoantigen‐specific B cells could be complementary. We used a streptavidin‐biotin coupling system in this study; however, an alternative system needs to be used to develop an antibody‐mediated cytotoxicity engager compatible with clinical use.

In conclusion, our study demonstrates that selective depletion of anti‐αECD‐antibody secreting cells, achieved via an anti‐hCD38/αECD‐ACE in a humanized mouse model of MG, effectively controls MG symptoms while preserving anti‐hAChR βECD control cells. This approach, therefore, represents a promising treatment strategy for autoimmune diseases characterized by antibody‐mediated pathology, as it specifically targets the disease‐mediating autoantibody‐producing hCD38^+^ cells. Importantly, it not only depletes short‐lived plasma cells in inflamed tissues but also targets treatment‐resistant long‐lived plasma cells in the bone marrow, potentially reducing disease recurrence. Furthermore, the treatment maintains protective immunity, suggesting a reduced risk of side effects compared with broad immunosuppression. Therefore, this novel therapeutic approach is leading the way toward safe and autoantigen‐specific treatments of autoimmune diseases.

## 4 Data Limitation and Perspectives

A limitation of this study is the focus on a mouse model, which does not fully recapitulate the complexity and heterogeneity of the human autoimmune disease myasthenia gravis. In particular, proliferation of antibody‐secreting hybridoma cells results in a narrow therapeutic window and limits long‐term assessment of treatment durability and regeneration of pathogenic plasma cells from precursor compartments. Another limitation is the potential underestimation of treatment effects due to the contribution of hCD38‐negative hybridoma cells, which are not targeted by the anti‐hCD38/αECD‐ACE.

In vivo experiments were performed in NSG‐Hc^1^ mice to overcome the known C5 complement deficiency of parental NSG mice. While NSG‐Hc^1^ mice retain functional complement activity, they lack adaptive immunity and exhibit severely impaired immune effector function [14], rendering NK cell– and T cell–mediated mechanisms unlikely. Consequently, the depletion efficiency observed in the current study may underestimate the full therapeutic potential of the approach, as antibody‐dependent cellular cytotoxicity (ADCC) cannot contribute under these conditions. Accordingly, complement‐dependent cytotoxicity is presumed to represent the predominant mechanism of plasma cell depletion, consistent with the complement‐mediated cytotoxic activity demonstrated in vitro [12].

While a detailed comparison with established CD19‐ and BCMA‐targeted therapies is provided in the discussion, the present strategy was evaluated in an immune‐restricted setting and therefore requires validation in immunocompetent, clinically relevant models to fully assess its translational potential. All animal experiments were conducted in accordance with German animal welfare regulations and the 3R principles. Ethical constraints limited animal numbers and experimental scope, potentially reducing statistical power while ensuring compliance with rigorous ethical standards.

## 5 Materials and Methods

### 5.1 Protein, Cell Lines, and Anti‐hCD38/αECD‐ACE

#### 5.1.1 Production of Recombinant α1‐ECD (hAChR αECD; αECD)

The extracellular domain (ECD) of the hAChR α1 subunit, mutated to have its Cys‐loop exchanged for that of the acetylcholine binding protein from *Lymnaea stagnalis*, was expressed in the yeast *Pichia pastoris* as a soluble secreted polypeptide. It was purified using a C‐terminal 6xHis tag through metal affinity chromatography, followed by size exclusion chromatography, as previously described [28].

#### 5.1.2 Production of Anti‐Human CD38 Antibody (anti‐hCD38)

The variable regions (VH and VL) of the anti‐hCD38 antibody were derived from Sanofi's proprietary sequences. The constant Fc domain sequence, which includes NNAS mutations [29], is utilized in the anti‐hCD38 antibody to neutralize Fc‐mediated cytotoxic activities. The anti‐hCD38 antibody with inactive NNAS IgG1 Fc has been produced in CHO cells (Evitria, Switzerland). To ensure the inactivity of the antibody, we conducted an in vitro cytotoxicity assay comparing it to daratumumab (Janssen). Our results showed no complement activity with the anti‐hCD38 containing an inactive Fc region, whereas daratumumab demonstrated significant cytotoxicity (unpublished).

#### 5.1.3 Preparation of Anti‐hCD38/αECD—Antibody‐Mediated Cytotoxicity Engager (Anti‐hCD38/αECD‐ACE)

The anti‐hCD38/αECD‐ACE was designed as a construct with two components: (a) an anti‐hCD38 antibody that labels all plasma cells, including both protective and autoreactive cells, and (b) the αECD antigen used to select only anti‐αECD‐specific plasma cells. In this study, streptavidin‐biotin conjugation was employed for protein‐conjugation. The anti‐hCD38 antibody with inactive NNAS IgG1 Fc sequence was coupled with streptavidin (SA) using the LYNX streptavidin antibody conjugation kit (Bio‐Rad). The hAChR αECD antigen was linked to biotin using the LYNX Rapid Plus Biotin Antibody Conjugation Kit (Bio‐Rad) following the manufacturer's protocol. For the coupling of the antibody with the antigen, anti‐hCD38‐SA and αECD‐biotin were mixed in a molar ratio of 1:6 and incubated for at least 15 min at room temperature before use. Integrity of the anti‐hCD38/αECD‐ACE was assessed by ELISA and flow cytometry.

### 5.2 Cell Lines and hCD38‐Transfection

Mouse anti‐hAChR αECD‐secreting hybridoma cells (clone D6) and mouse anti‐hAChR βECD‐secreting hybridoma cells (clone B3/2) were generously donated by the myasthenia gravis community in Berlin. Isotype determination performed in this study identified the anti‐αECD antibody (D6) as IgG2b and the anti‐β ECD antibody (B3/2) as IgG1.

To generate a humanized model, we transfected mouse hybridoma cells with hCD38 using two different methods, one containing GFP. The hCD38‐expressing D6 cell line was generated via electroporation with a vector harbouring the hCD38 sequence (ENA|BAA18964|BAA18964.1 Homo sapiens (human) CD38), flanked by pT4‐sequences [30] (designed in silico and produced by BioCat) and SB100X RNA [31]. The SB100X RNA production was performed with the HighYield T7 RNA Synthesis Kit (Jena Biosciences) with the use of a vector containing the SB100X sequence flanked by a 5′HBB UTR, a 3′HBB UTR, and a 90 nt poly‐A tail, which was linearized via Spel and Bbsl restriction enzymes (NEB) and purified with Mag‐Bind Total Pure NGS magnetic beads (Omega Biotek) before RNA synthesis. The generated RNA was purified using the same beads and stored at −80°C. Electroporation of the cells was performed with a Lonza 4D Nucleofector with the P3 primary cell kit (Lonza) and program CA‐158. Transfected cells were enriched by staining with anti‐hCD38‐AF488 (Biolegend, clone HIT2) and selection using the FITC positive selection Kit (Stemcell), followed by sorting for hCD38^hi^ cells using a MAQSQuant Tyto Cell Sorter (Miltenyi). A limiting dilution of the cells was conducted to generate monoclonal subcell lines. The procedure was performed by MDCell Berlin (Max Delbrück Centre, Boost Innovation Lab).

B3/2 hybridoma cell line was transfected with linearized pCDH‐MSCV‐hCD38‐EF1α‐GFP+Puro plasmid (a gift from Jianjun Zhao (Addgene plasmid #134936; http://n2t.net/addgene:134936; RRID:Addgene_134936) encoding *hCD3*8 using Neon (Thermo Scientific) electroporation system (1300 V, 1 pulse, 20 ms). After transfection, cells were incubated overnight at 37°C, 5% CO_2_ in RPMI‐1640, 10% FCS, P/S. The next day, puromycin (10 µg/mL) was added to the culture. Seven days later, the transfected cells were cloned by limited dilution and analyzed for GFP and hCD38 expression using flow cytometry.

## 6 Mouse Experiments

### 6.1 Mice

Female NSG‐Hc^1^ (NOD.Cg‐*Hc^1^ Prkdc* ^scid^ *Il2rg* ^1Wjl^/*SzJ*) mice were obtained from the Jackson Laboratory (JAX stock #030511) and kept at the animal facility of German Rheumatology Research Centre Berlin under specific pathogen‐free conditions. All experiments were performed in accordance with German laws for animal protection and approved by the local authorities. Mice were age‐matched in each experiment.

All animal numbers were determined in consultation with a statistician to ensure sufficient statistical power while adhering to animal protection law and the 3R principles. Once robust and consistent results were obtained, further repetitions were avoided to prevent unnecessary animal use.

### 6.2 Clinical Scoring of Mice

Grip strength loss, reduced mobility, and hunched posture are the main MG‐like symptoms observed in MG‐induced mice. In this study, we measured the grip strength (maximal muscle force of the forepaws) using a grip strength meter (Bioseb, France) before and after one minute of repeated grasping exercise while being pulled over a grid.

The MG‐like clinical score was classified into four MG grades based on the following criteria: score 0, no symptoms; score 1, no signs at rest time of mice but grip strength loss after exercise; score 2, mild hunched posture, reduced mobility at rest time and loss of grip strength before and after exercise; score 3, severe hunched posture and paralysis. The animals were considered to be sick when they reached score 1 (i.e., when they displayed altered movements) [32, 33]. According to animal welfare regulations, mice that lost more than 10% of their initial body weight were kept for a maximum of seven additional days before being sacrificed. Animals that displayed an MG score of 2 were kept for a maximum of 10 days after diagnosis. An MG score of 3 or body weight loss of more than 20% were considered an immediate endpoint criterion.

### 6.3 Enzyme‐Linked Immunosorbent Assay (ELISA) Assessments

#### 6.3.1 Analysis of the Anti‐hCD38/αECD‐ACE Integrity

ELISA plates (Sarstedt) were coated with mouse‐anti‐hAChR αECD (5 µg/mL; clone D6, DRFZ) or mouse‐anti‐hAChR βECD (5 µg/mL; clone B3/2, DRFZ), blocked with PBS/3% BSA, and incubated with the sample for 2 h at 37°C. For detection, we used a goat‐anti‐human IgG‐AP antibody (0.3 µg/mL; Southern Biotech) for 1 h at 37°C. Plates were developed using p‐nitrophenyl phosphate (PNPP) substrate (Thermo Scientific) for 30 min. Signal was measured at 405 nm with a SpectraMax i3x ELISA reader (Molecular Devices).

#### 6.3.2 Detection of Antibody Levels in Mouse Serum

ELISA plates were coated with either goat‐anti‐mouse IgG (5 µg/mL; Southern Biotech), hAChR αECD (10 µg/mL), or hAChR βECD (5 µg/mL; MyBiosource MBS717559). For detection, a goat‐anti‐mouse IgG‐AP antibody (0.3 µg/mL; Southern Biotech) was used. For the detection of αECD‐specific antibodies via their subclass, a goat‐anti‐mouse IgG2b‐HRP antibody was used (0.1 µg/mL; Southern Biotech). Mouse‐anti‐hAChR αECD or mouse‐anti‐hAChR βECD antibodies were used as a standard for the calculation of the respective antibody level.

## 7 Quantitative Analysis of Mouse C3 and C5a Complement Proteins in Mouse Serum

Sera from mice were collected, and C3 and C5a measurement was performed using ELISA kits (Biomatik, Canada) according to the manufacturer's protocol.

### 7.1 Flow Cytometric Analysis

#### 7.1.1 In Vitro and in Vivo Labeling of hCD38‐Transfected Hybridoma Cells with Anti‐hCD38/αECD‐ACE

The anti‐hCD38 coupled to SA of the anti‐hCD38/αECD‐ACE was detected with anti‐human Ig‐light chain κ and λ (clones MHK‐49 and MHL‐38, respectively) and anti‐SA (clone 3A20.2). The biotinylated hAChR αECD of the anti‐hCD38/αECD‐ACE was detected by SA (Biolegend) and anti‐hAChR αECD (clone D6, DRFZ).

#### 7.1.2 The Engrafted Hybridoma Cells Detection in NSG‐Hc^1^ Mice

Single cell suspensions of bone marrow and spleen were blocked with anti‐FcγR (clone 2.4G2/75, DRFZ). Extracellular staining of hCD38 and mCD138 was used to detect hCD38^−^ and hCD38^+^ transfected D6 (GFP^−^) and B3/2 (GFP^+^) hybridoma cells.

Detection of viable cells was performed with DAPI (Sigma Aldrich). Antibodies, if not indicated otherwise, were purchased from BioLegend. The samples were acquired at a FACSCanto II cytometer (BD Biosciences), and data were analyzed with FlowJo Software Version 10.2.1 (Tree Star Inc.).

### 7.2 Statistical Analysis

All analyses were performed with GraphPad Prism software version 10.2.1. A nonparametric, two‐tailed Mann–Whitney *U* Test was used to compare data from the treated group with controls. For the comparison of antibody titers, disease score, and body weight between the treated and the vehicle group, two‐way ANOVA or restricted maximum likelihood model with Geisser‐Greenhouse correction for datasets with missing values was performed. Survival curves were compared via the log‐rank test. *p *< 0.05 was regarded as statistically significant, with *p < 0.05, **p < 0.01, and ***p < 0.001.

## Author Contributions

Laleh Khodadadi generated, analyzed, and verified the data, supervised the project, conceptualized the research, established the protocols, made the figures, and wrote the manuscript. Deborah Puppe generated, analyzed, and verified the data, conceptualized the research, established the protocols, made the figures, and wrote the manuscript. Dilara S. Cirillo generated, analyzed, and verified the data. Carolina Martinez‐Cingolani, Andreas Pelz, Siegfried Kohler, Qingyu Cheng, Tobias Alexander, and Andreas Radbruch conceptualized the research. Jens Klotsche contributed to the statistical analysis. Konstantinos Lazaridis provided the ECD protein. Michael Fichtner, Jan Pille, Andrey Kruglov, and Marina Bondareva transfected the cell lines. Andreas Meisel and Falk Hiepe supervised the project and conceptualized the research. All other authors read, edited, and approved the manuscript.

## Conflicts of Interest

Carolina Martinez‐Cingolani is an employee of Sanofi and may hold shares and/or stock options in the Company. The remaining authors declare no conflicts of interest.

## Supporting information




**Supporting File**: eji70166‐sup‐0001‐SuppMat.pdf.

## Data Availability

The data supporting the findings of this study are available from the corresponding authors upon reasonable request.
