# Supplementary material for: Selective Depletion of Autoreactive Plasma Cells as a Novel Strategy to Treat Acetylcholine Receptor Antibody‐Positive Myasthenia Gravis
Source: Eur J Immunol. 2026 Mar 29;56(4):e70166. doi: 10.1002/eji.70166 (PMC13033962; doi:10.1002/eji.70166)
Supplement: Supplementary file 1 — Supporting File: eji70166‐sup‐0001‐SuppMat.pdf. [file EJI-56-e70166-s001.pdf]

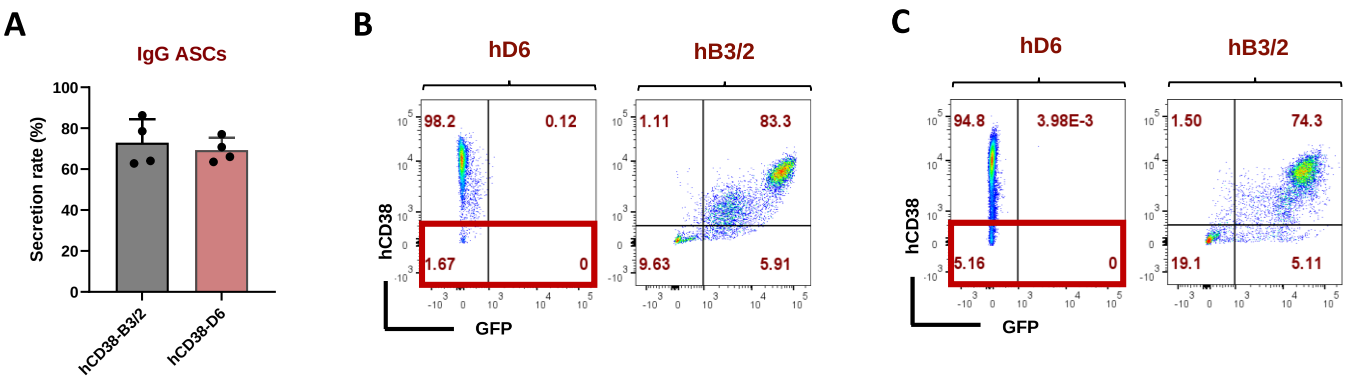


**Suppl. Fig 1. *In vitro* check of hybridoma cells prior to adoptive transfer into NSG-Hc[1] mice.**

**(A)** Secretion of hD6 and hB3/2 hybridoma cells seperately analyzed by ELISPOT. The bars represent mean of secretion rate (%) per cell line. **(B & C)** Representative FACS dot blot for hCD38 and GFP expression in hCD38 transfected D6 and B3/2 hybridoma cells before transfection into NSG-Hc1 mice for first round and second round of specific depletion experiments, respectively.

hCD38-D6; human CD38 transfected into mouse anti-human AChR αECD secreting D6 (GFP-) hybridoma cells, hCD38-B3/2; human CD38 transfected into mouse anti-human AChR βECD secreting B3/2 (GFP+) hybridoma cells.

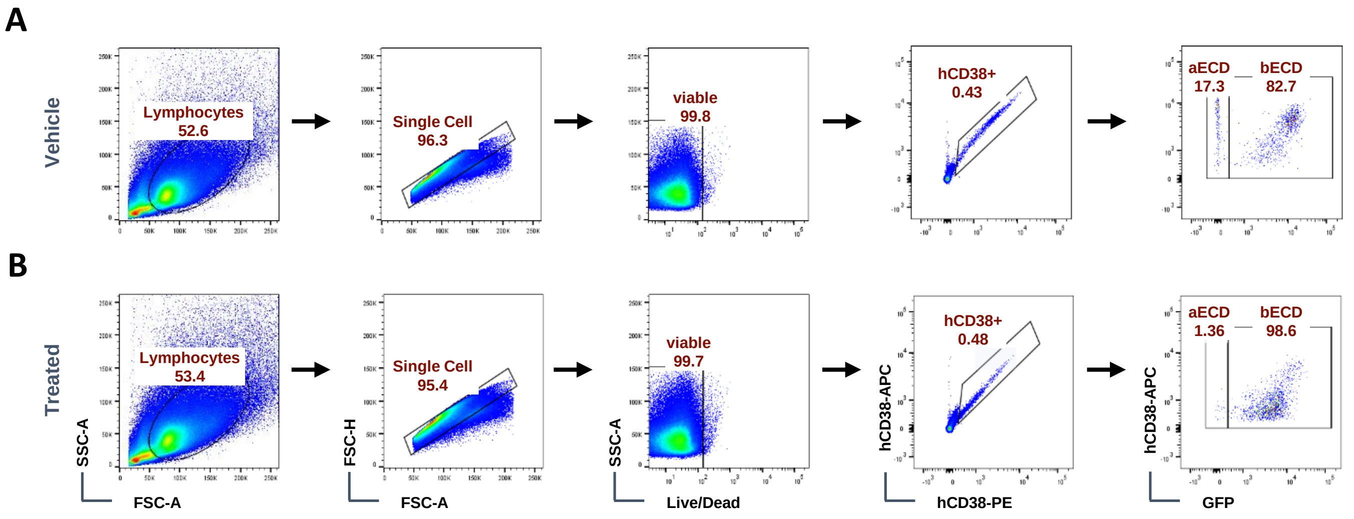


**Suppl. Fig 2. Gating strategy for the detection of hCD38+ hybridoma cells in humanized NSG-Hc [1] MG mice.**

Representative FACS dot blot for detection of βECD-specific hCD38-B3/2 (GFP+) and αECD-specific hCD38-D6 (GFP-) cells in **(A)** Vehicle-treated mice and **(B)** mice treated with anti-hCD38/αECD-ACE.

hCD38-D6; human CD38 transfected into mouse anti-human AChR αECD secreting D6 (GFP-) hybridoma cells, hCD38-B3/2; human CD38 transfected into mouse anti-human AChR βECD secreting B3/2 (GFP+) hybridoma cells, anti-hCD38/αECD-ACE; anti-hCD38/αECD-antibody-mediated cytotoxicity engager.

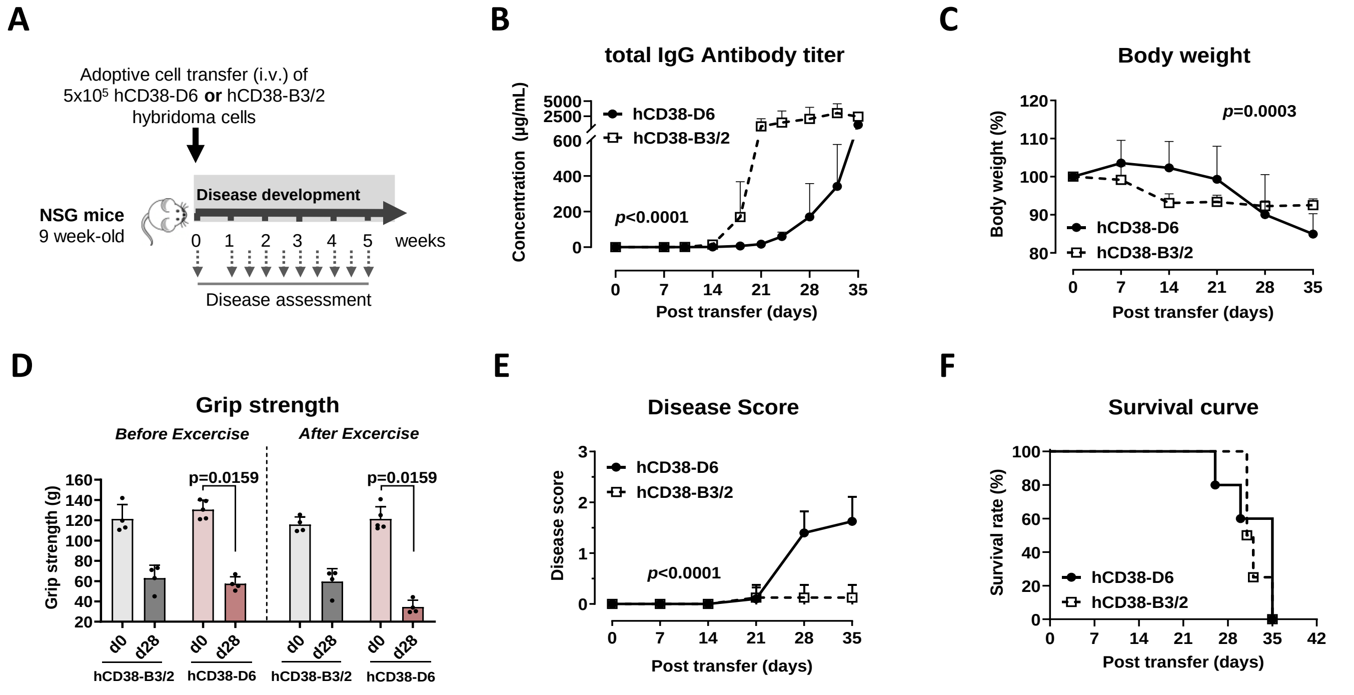


**Suppl. Fig 3. Humanized mouse model of anti-AChR antibody-positive generalized myasthenia gravis (NSG MG mice).**

**(A)** Animal experiment design. Immunodeficient NSG mice were divided into two groups and $5x10^5$ of hCD38-D6 (GFP-) (n=5) or hCD38-B3/2 (GFP+) (n=4) hybridoma cells were transferred intravenously into mice. Induction of NSG MG mice was assessed by **(B)** anti-IgG antibody concentration secreted by hybridoma cells (µg/ml), **(C)** body weight (%), **(D)** grip strength test (g), **(E)** disease score measurement and **(F)** survival curve of each group of mice. Values are mean ± SD and comprise the number of animals alive at the respective timepoints; *P <0.05; **P<0.01, ***P<0.001, analyzed by Mann Whitney U test, mixed effect analysis or Log-rank test.

hCD38-D6; human CD38 transfected into mouse anti-human AChR αECD secreting D6 (GFP-) hybridoma cells, hCD38-B3/2; human CD38 transfected into mouse anti-human AChR βECD secreting B3/2 (GFP+) hybridoma cells.

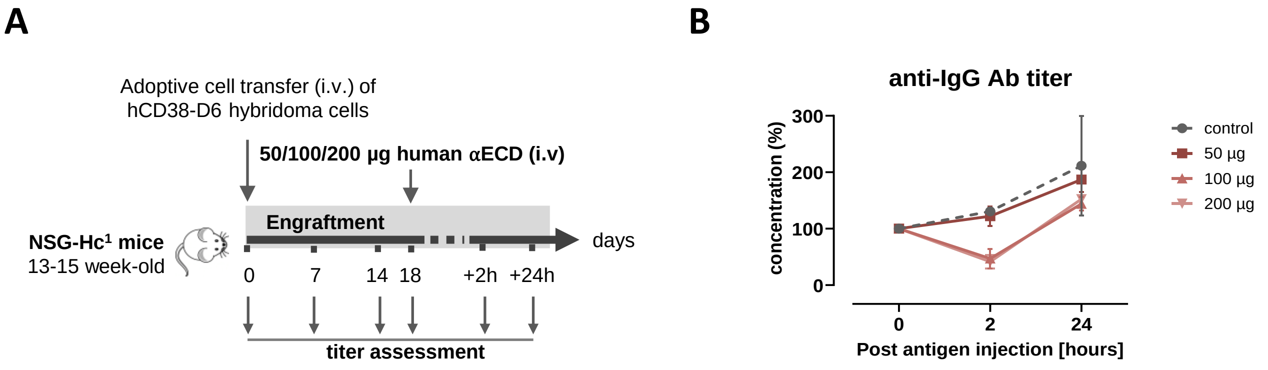


**Suppl. Fig 4. Short term titer reduction by antigen injection in humanized NSG-Hc [1] MG mice.**

**(A)** Animal experiment design. Immunodeficient NSG-Hc[1] mice were injected with $5x10^5$ of hCD38-D6 (GFP-) hybridoma cells. Engraftment of cells was observed by antibody titer assessment. On day 18 after cell transfer, 50, 100 or 200 µg of human αECD were injected intraveneously (n=3 each). A control group received PBS (n=2). **(B)** The titer reduction was assessed by anti-IgG antibody concentration in the blood before, 2h and 24h after antigen injection and displayed as relative reduction per group compared to antibody concentration before antigen injection (%). Values are mean ± SD; analysed by Mann Whitney U test.

hCD38-D6; human CD38 transfected into mouse anti-human AChR αECD secreting D6 (GFP-) hybridoma cells.
